# Macrolunula: Case Reports of Patients with Trauma-associated Enlarged Lunula and a Concise Review of this Nail Finding

**DOI:** 10.7759/cureus.2998

**Published:** 2018-07-18

**Authors:** Pallavi Basu, Philip R Cohen

**Affiliations:** 1 School of Medicine, University of California San Diego, La Jolla, USA; 2 Dermatologist, San Diego Family Dermatology, San Diego, USA

**Keywords:** enlarged, hyperthyroidism, injury, leprosy, lunula, macrolunula, matrix, nail, scleroderma, trauma

## Abstract

The lunula refers to the visible portion of the distal nail matrix that extends beyond the proximal nail fold. Macrolunula, or enlarged lunula, is not only a physiologic variant but also has been associated with a variety of local and systemic disorders. Macrolunula has been described in congenital conditions including hereditary onycho-osteodysplasia, neoplasms such as superficial acral fibromyxoma, as well as iatrogenic causes as in the topical administration of hydrocortisone; it can also occur in systemic disorders including hyperthyroidism, ischemia, leprosy, and scleroderma. While macrolunula has been described in self-induced trauma secondary to habit-tic deformity, we observe in this case report that any mechanism of trauma to the nail unit may produce enlargement of the lunula.

## Introduction

The lunula is the white crescent-shaped proximal portion of the nail plate. It is more prominent on the thumbs and great toes and less obvious or absent on the other digits [1]. Macrolunula, exaggerated length of this portion of the nail plate, may occur as a normal variant or be associated with nail unit disorders or systemic conditions [2]. Three patients with macrolunula are described and the conditions associated with this nail feature are discussed.

## Case presentation

**Figure 1:**
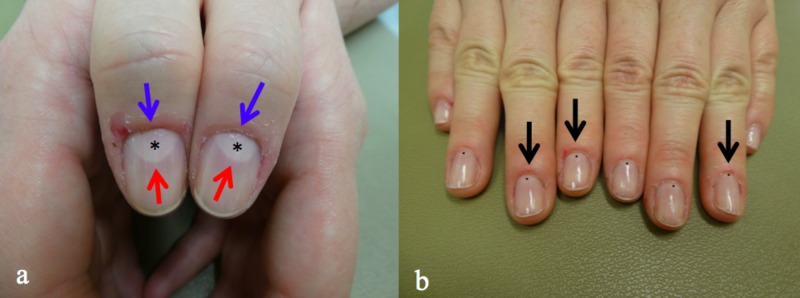
Macrolunula caused by trauma to the cuticle (eponychium) A 49-year-old woman with a) markedly enlarged lunula (*) of bilateral thumbs visible between the proximal nail fold (blue arrow) and remainder of the nail plate (red arrow) and b) mildly to moderately enlarged lunula (black dots) as well as inflammation of the cuticles (black arrows) on the other digits of both hands.

Case 1: A 49-year-old woman presented to the dermatologist for routine skin screening. Her past medical history was significant for fibromyalgia, hyperlipidemia, hypertension, and obstructive sleep apnea. She had also a history of actinic keratosis, basal cell carcinoma, and melanoma. Cutaneous examination showed dark pigmented lesions on her right scapula, right axilla, and left chest. Biopsy revealed benign nevi. Her skin examination also revealed ragged cuticles with erosions. In addition, the lunula were markedly enlarged on both thumbs (macrolunula). The lunula on some of her other fingers were also prominent (Figure 1). Additional history revealed that she habitually used the nails of one hand to pick at the cuticles (eponychium) of the other hand. Her macrolunula-associated characteristics are summarized in Table 1.

**Table 1:** Characteristics of patients with macrolunula

Case	Age, race, sex	Affected digits of hand	Associated etiology
1	49-year-old Caucasian woman	Multiple digits, bilateral	Trauma to the cuticle
2	58-year-old Caucasian woman	Left thumb	Trauma to the nail matrix
3	64-year-old Caucasian man	Right thumb	Trauma to the proximal nail fold

**Figure 2:**
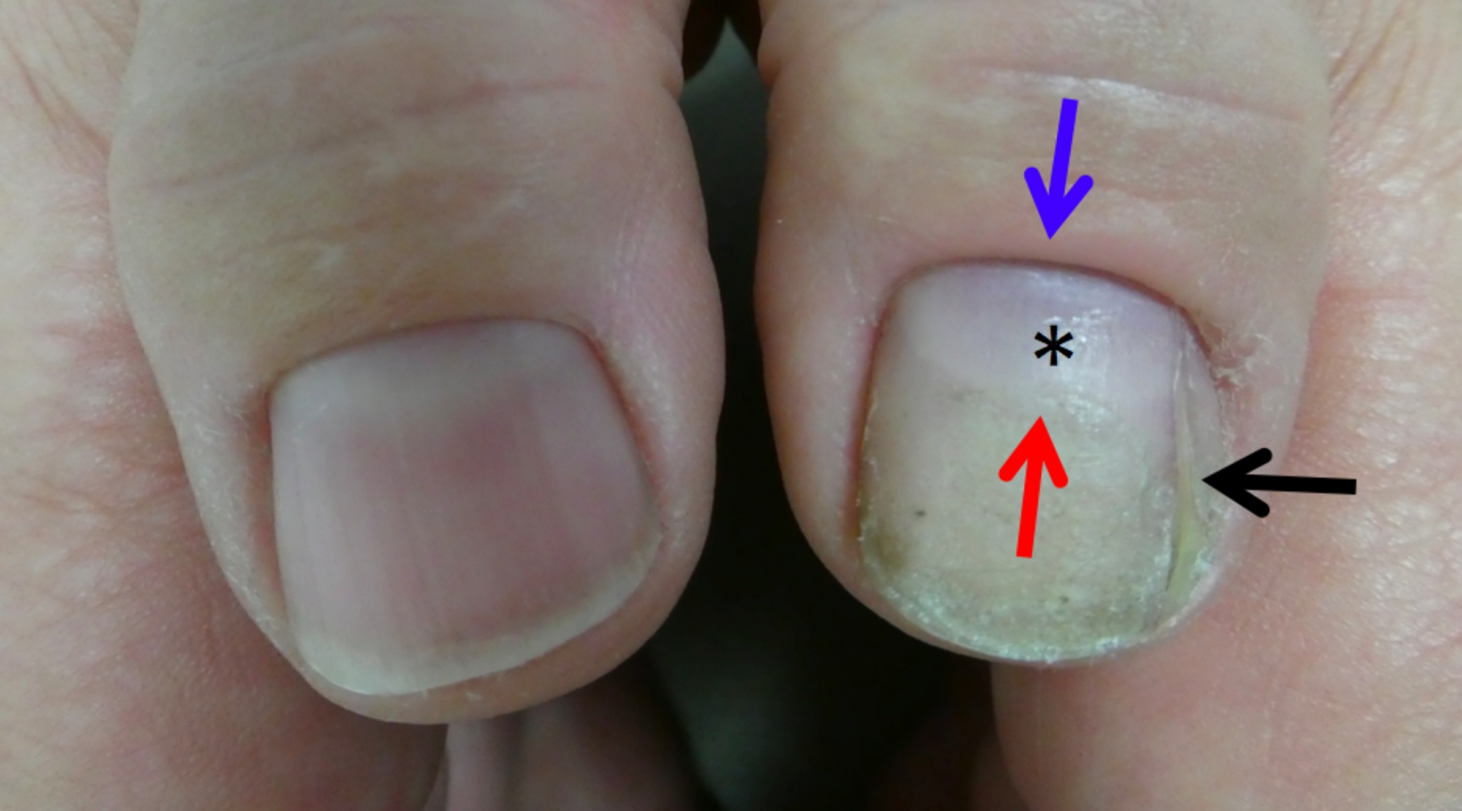
Macrolunula associated with trauma to the digit that resulted in nail matrix scar and nail plate deformity A 58-year-old woman with a split in the lateral portion of her left thumbnail extending from the proximal nail fold to the tip of the nail (black arrow) as well as enlargement of the lunula of the left thumb nail (*); clinically the macrolunula is demonstrated by the white-appearing region between the proximal nail fold (blue arrow) and the remainder of the nail plate (red arrow).

Case 2: A 58-year-old woman came in for evaluation of a lesion on her right thigh that had enlarged. She had a past medical history of arthritis. A cutaneous exam showed an ulcerated nodule on her right thigh. Biopsy showed benign prurigo nodularis. Examination of her nails showed a split in the lateral portion of her left thumbnail that extended from the proximal nail fold to the tip of the nail. The lunula of the left thumb was significantly enlarged (Figure 2). Additional history revealed that the finger had been caught in a car door when she was age 17, and the nail plate had subsequently split. Her macrolunula-associated characteristics are summarized in Table 1.

**Figure 3:**
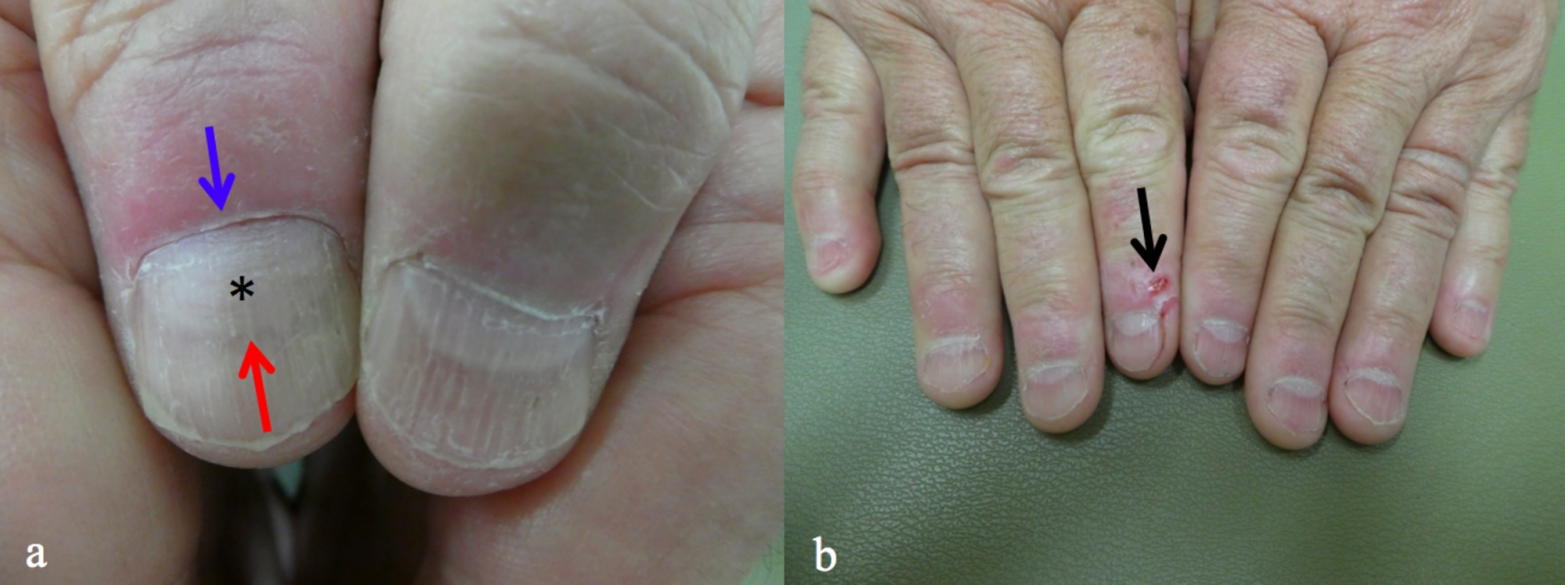
Macrolunula secondary to repetitive rubbing of the proximal nail fold A 64-year-old man with a markedly enlarged lunula (*) of the right thumb (a) that is visible between the proximal nail fold (blue arrow) and the remainder of nail plate (red arrow); there are erosions (black arrow) and altered nail folds on the other digits (b).

Case 3: A 64-year-old man came in for evaluation of a lesion on his left upper lip. His past medical history included gastroesophageal reflux disease, hyperlipidemia, and obstructive sleep apnea. Skin history included a prior basal cell carcinoma. Cutaneous examination revealed a plaque on his upper lip; a biopsy of the lesion diagnosed squamous cell carcinoma in situ. Cutaneous examination of the fingers also showed erosions and altered nail folds. Moreover, the lunula of his right thumb was markedly enlarged (Figure 3). Further history indicated that the patient often bites off the distal ends of his fingernails. He also habitually rubs the proximal nail fold of his right thumb. His macrolunula-associated characteristics are summarized in Table 1.

## Discussion

The nail plate is bordered by the lateral and proximal nail folds. The matrix is the growth center of the nail; it is partially covered by the proximal nail fold. The lunula is that portion of the nail matrix that extends beyond the proximal nail fold and clinically appears white [1].

The size and shape of the lunula may vary. The lunula may be small or absent or asymmetric when comparing multiple digits of each hand. In addition, the lunula of an individual digit or corresponding digits may be larger than average or exaggerated in size; this is referred to as macrolunula [2]. However, to date, a definition that precisely establishes the criteria for macrolunula - either in specific measurements or in proportion to the entire nail plate - does not exist.

Macrolunula has been associated with local disorders that directly affect the nail unit. In addition, tumors and systemic conditions can also result in macrolunula. These conditions are listed in Table 2 [3-13].

**Table 2:** Conditions associated with macrolunula CR: current report; C: case.

Condition	Reference
Congenital	3
Nail-patella syndrome	3
Habit-tic deformity	4
Median nail dystrophy	5
Medication side effect	6
Topical hydrocortisone	6
Normal variant	7
Superficial acral fibromyxoma	8
Systemic disorders	9-13
Hyperthyroidism	9
Leprosy	11-13
Scleroderma	10
Trauma	CR
Eponychium injury	CR, C1
Matrix injury	CR, C2
Proximal nail fold injury	CR, C3

Macrolunula can be a normal variant in patients from India [7]. It can also be associated with idiopathic conditions such as median nail dystrophy; this typically presents as small cracks or fissures extending laterally from the central canal or split toward the nail edge giving the appearance of an inverted fir tree [5]. Additionally, the application of hydrocortisone to the cuticle has been observed to result in enlarged lunula [6].

A congenital condition associated with macrolunula is nail patella syndrome, or hereditary onycho-osteodysplasia. Patients present with the tetrad of an absent patella, nail changes, iliac horn abnormalities, and elbow anomalies. The nail change that is considered to be pathognomonic for the disease is a triangular lunula that appears enlarged [3].

Macrolunula can also be seen in acquired nail dystrophies associated with trauma to the nail plate. This trauma to the nail plate may be self-induced, albeit unintentional, as in habit-tic deformity in which the patient uses one digit to rub a localized area of the proximal nail fold of another digit, altering the matrix below. In these individuals, the discovery of the enlarged lunula may be a subtle indication of underlying psychiatric illness, especially body-focused repetitive behaviors [4].

Exogenous injury to the nail matrix may also result in macrolunula. For e.g., biting, picking, and/or rubbing the proximal nail fold, as illustrated by two of our patients, traumatized the underlying nail matrix and resulted in the development of macrolunula. In one of our patients, traumatic injury to her thumb by a car door, causing compression and scar to the nail's matrix, likely resulted in enlargement of the lunula.

Superficial acral fibromyxoma is a benign soft tissue tumor located in the acral areas, particularly the periungual and subungual areas that may present with triangular macrolunula [8]. In one case study, a 50-year-old woman presented with three years of progressive painless enlargement of the nail base of her left thumb with a very wide, triangular-shaped lunula on exam and no underlying bone involvement on phalangeal radiographs. After avulsion of the nail plate, incision of the matrix revealed a pink, translucent nodular lesion of 2 centimeters in diameter. Histopathological examination revealed a dermal nodular tumor, characterized by proliferation of fusiform cells without atypia, within a myxoid stroma rich in dilated capillaries and mast cells [8].

Systemic conditions may be associated with enlarged lunula. These include hyperthyroidism, leprosy, and scleroderma [9]. Investigators evaluated 39 patients with connective tissue diseases including dermatomyositis, scleroderma, Sjogren’s syndrome, and systemic lupus erythematosus. 16 of the 39 patients (41%) had scleroderma. Among these patients, one (6.25%) was found to have macrolunula [10].

Diffusion of the lunula, also referred to as pseudomacrolunula, can mimic macrolunula; however, in true macrolunula the nail plate itself is involved, whereas in pseudomacrolunula, the subungual tissue is affected [11]. In leprosy, both pseudomacrolunula and true macrolunula may be noted; pseudomacrolunula may represent a common and early sign of the disease [11-13]. In addition, pseudomacrolunula may also be observed in conditions causing ischemia to the nail unit [14].

## Conclusions

Macrolunula describes when the portion of the nail matrix extending beyond the proximal nail fold is enlarged. Several associated congenital, acquired, physiologic, and systemic etiologies for this clinical finding have been observed. In addition, local trauma to the nail unit can also be a common cause of macrolunula. The detection of macrolunula may be a clue to an underlying body-focused repetitive behavior or a benign soft tissue tumor. Alternatively, this nail finding may be associated with hereditary conditions and systemic disorders.
